# Inspiratory effort estimated by airway occlusion pressure in the presence of intrinsic positive end-expiratory pressure

**DOI:** 10.1186/s13054-026-06039-0

**Published:** 2026-04-22

**Authors:** Ran Gao, Mattia Docci, Andrea Coppadoro, Roberto Brito, Ewan C. Goligher, Giacomo Bellani, Laurent Brochard

**Affiliations:** 1 Keenan Centre for Biomedical Research, Li Ka Shing Knowledge Institute, Unity Health Toronto, Toronto, ON Canada; 2 Interdepartmental Division of Critical Care Medicine, University of Toronto, Toronto, ON Canada; 3 Surgical Intensive Care Unit, Emergency and Critical Care Medical Center, Clinical and Research Center on Acute Lung Injury, Beijing Shijitan Hospital, Capital Medical University, Beijing, China; 4 Critical Care Department, St. Michael’s Hospital, Unity Health Toronto, Toronto, ON Canada; 5 Anesthesia and Critical Care Department, IRCCS San Gerardo, Monza, Italy; 6 Departamento de Medicina Interna Norte, Facultad de Medicina, Universidad de Chile, Santiago, Chile; 7 Department of Physiology, University of Toronto, Toronto, ON Canada; 8 Division of Respirology, Department of Medicine, University Health Network, Toronto, ON Canada; 9 Toronto General Hospital Research Institute, Toronto, ON Canada; 10 Centre for Medical Sciences-CISMed, University of Trento, Trento, Italy; 11 Department of Anesthesia and Intensive Care, Santa Chiara Hospital, Trento, Italy

**Keywords:** Intrinsic positive end-expiratory pressure, Airway occlusion pressure, Pressure support ventilation

## Abstract

In patients with auto- or intrinsic positive end-expiratory pressure (PEEPi), a portion of the inspiratory effort is spent to overcome PEEPi before lung inflation begins. End-expiratory whole-breath airway occlusion pressure (ΔPocc) is a useful tool to estimate inspiratory muscle pressure (ΔPmus) but it may not capture this component. We retrospectively analyzed data from patients with various degrees of PEEPi enrolled in a study who underwent PEEP titration during pressure support ventilation (PSV) with esophageal pressure (Pes). Inspiratory effort was partitioned into the effort generated before inflation and the effort required for ventilator triggering and inflation. ΔPmus, Pes swing (ΔPes) and dynamic transpulmonary pressure (ΔP_L, dyn_) were both measured and predicted from ΔPocc using established conversion factors. Linear mixed-effects models were used to evaluate the effect of PEEPi on estimation accuracy. The results showed that (1) PEEPi values ranged from mean (± SD) of 4.7 (± 5.0) to 1.2 (± 0.7) cmH_2_O from PEEP 2 to 14 cmH_2_O; (2) ΔPocc does not represent the total inspiratory effort when PEEPi or relaxation of active expiration is present, and is limited to the triggering and inflation components; (3) when the airway pressure following ΔPocc exhibits a visible stable plateau, PEEPi may be estimated under selected waveform conditions on the ventilator screen; (4) Baydur’s maneuver for esophageal balloon calibration might be biased in case of PEEPi or active expiration; (5) the predicted estimate of ΔP_L, dyn_ appears acceptable at group level but can lack precision. Conclusion: this proof-of-concept study shows that ΔPocc may underestimate inspiratory effort in case of PEEPi. When a plateau airway pressure can be observed in the end of ΔPocc, it seems possible to estimate PEEPi noninvasively.

**Supplementary Information:**

The online version contains supplementary material available at 10.1186/s13054-026-06039-0.

## Background

Measuring inspiratory effort is important for delivering a lung and diaphragm protective ventilation [1]. The end-expiratory occlusion (EEO) maneuver has been proposed as a simple tool to measure the whole-breath airway occlusion pressure (ΔPocc) as an index of inspiratory effort [2]. Studies in patients with hypoxemic respiratory failure have shown that ΔPocc can predict the respiratory muscle pressure (ΔPmus) as well as esophageal pressure swing (ΔPes), which could be applied to calculate dynamic transpulmonary pressure (ΔP_L, dyn_ ) [2–4].

Intrinsic end-expiratory positive pressure (PEEPi) or auto-PEEP is common in mechanically ventilated patients, mostly at low values. When PEEPi is present, a portion of the inspiratory effort is spent overcoming PEEPi during the last part of expiration before triggering the ventilator [5]. Accurately measuring PEEPi during pressure support ventilation (PSV) is challenging. EEO is unreliable when the respiratory muscles are not fully relaxed and expiratory muscle activity is present [6, 7].

We hypothesized that ΔPocc may not reflect the total effort in the presence of PEEPi, because the pressure required to overcome PEEPi occurs before airway is occluded. ΔPocc may primarily reflect the effort required to inflate the respiratory system after triggering the ventilator. We aimed to verify this hypothesis and explore the implications of PEEPi for three clinical applications of ΔPocc: noninvasive assessment of effort in case of PEEPi, Baydur maneuver for esophageal pressure calibration [8], and the estimation of ΔP_L, dyn_.

## Methods

### Population

We retrospectively analyzed data from a previously published study [9], which enrolled intubated patients receiving PSV and having a clinical suspicion of PEEPi (characteristics of the patients are given in *Supplemental Table 1)*. The dataset included respiratory mechanics measurements from ten patients, with esophageal pressure (Pes) and occlusions available in nine; gastric pressure (Pga) was available in six subjects. In order to modify PEEPi, each patient underwent a standardized PEEP titration protocol from 2 to 14 cmH₂O in 2 cmH₂O increments with constant PSV. EEO was performed at every PEEP level.

### Analysis

**Fig. 1:**
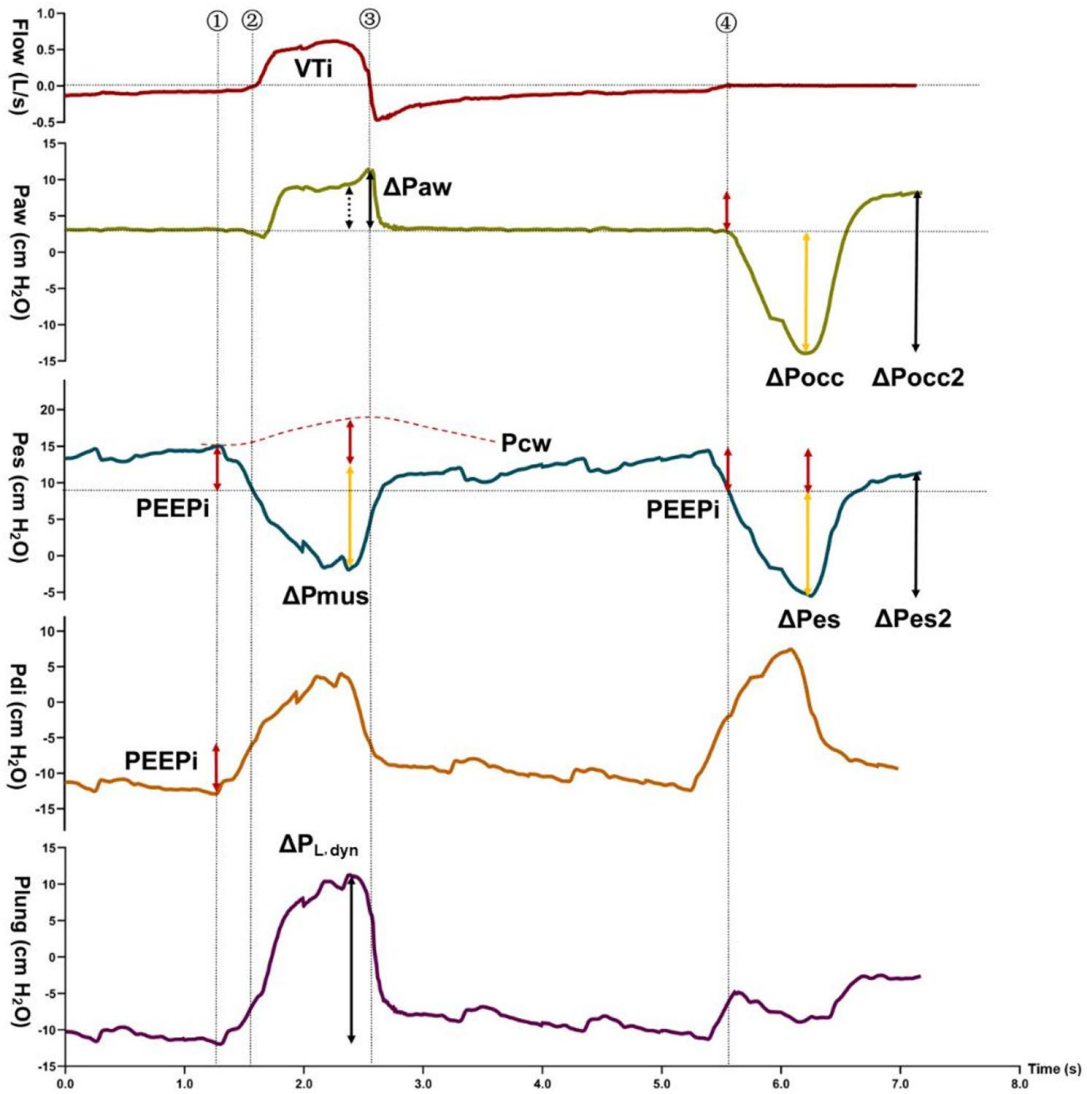
Description of the different components included in the analysis. Representative tracing of Flow, airway pressure (Paw), esophageal pressure (Pes), trans-diaphragmatic pressure (Pdi), and trans-pulmonary pressure (Plung) from top to bottom. Dashed lines (①–④) indicate: ① onset of inspiratory effort, ② onset of the trigger phase (last negative flow value), ③ cycling and ④end-expiratory occlusion. Compared with ΔPes_before inflation_, ΔPdi_before inflation_ more accurately represents the effort needed to overcome PEEPi and corrects for expiratory muscle relaxation (red arrows on the Pes and Pdi tracing). ΔPmus was calculated as the maximal difference between the chest wall recoil pressure and the Pes swing (ΔPmus is represented by the combined lengths of the red and yellow arrows; Pcw is the red dashed line on the Pes tracing). Inspiratory effort is partitioned into the effort before inflation (or before EEO, showed by red arrows on the Pes), which mainly reflects the effort required to overcome PEEPi, and the effort during inflation (or after EEO, showed by yellow arrows on the Pes tracing). The difference between the maximum upward Paw deflection and ΔPocc during EEO could be used to estimate PEEPi in Plateau pattern (ΔPocc 2 – ΔPocc, showed by red arrows on the Paw tracing). Ratio 1 is calculated as ΔPocc / ΔPes which is biased in presence of PEEPi, whereas the modified Ratio 2 uses ΔPocc / ΔPes_after EEO_ to eliminate PEEPi-related bias, and the modified Ratio 3 uses ΔPocc2 / ΔPes2 to incorporate all PEEPi-related influences. The *reference* ΔP_L, dyn_ is the instantaneous difference between Paw and Pes, while ΔPaw + ΔPes may be overestimated because ΔPaw and ΔPes are often not fully synchronized

1) The *reference* ΔPmus was measured from Pes tracing as the largest difference between chest wall recoil pressure (Pcw) and Pes swing. The *predicted* ΔPmus was 0.75 × ΔPocc and the *predicted* ΔPes was 0.66 × ΔPocc [2, 10].

2) To distinguish the components of effort, we separated *reference* ΔPmus into (Fig. 1) the negative pleural pressure generated before inflation (ΔPes_before inflation_) and the effort required to trigger ventilator and inflate the respiratory system (ΔPmus_inflation_) [11]. ΔPes_before inflation_ was the decrease in Pes required to abruptly bring expiratory flow zero (or to the closest value). As previously shown [12], part of this decay can be due to expiratory muscle relaxation, and part by active inspiratory effort. Therefore, in the six patients with reliable Pga measurement, the effort used to overcome PEEPi (ΔPdi_before inflation_) was calculated as the corresponding decrease in transdiaphragmatic pressure (Pdi). *Reference* PEEPi was defined as either ΔPes_before inflation_ (might be influenced by Pga drop, *n* = 9) or ΔPdi_before inflation_ (PEEPi, *n* = 6).

### Primary endpoint

The primary endpoint was to compare the difference between the measured ΔPmus *reference* and the ΔPmus *predicted* from ΔPocc in case of PEEPi.

### Secondary endpoints

**Fig. 2:**
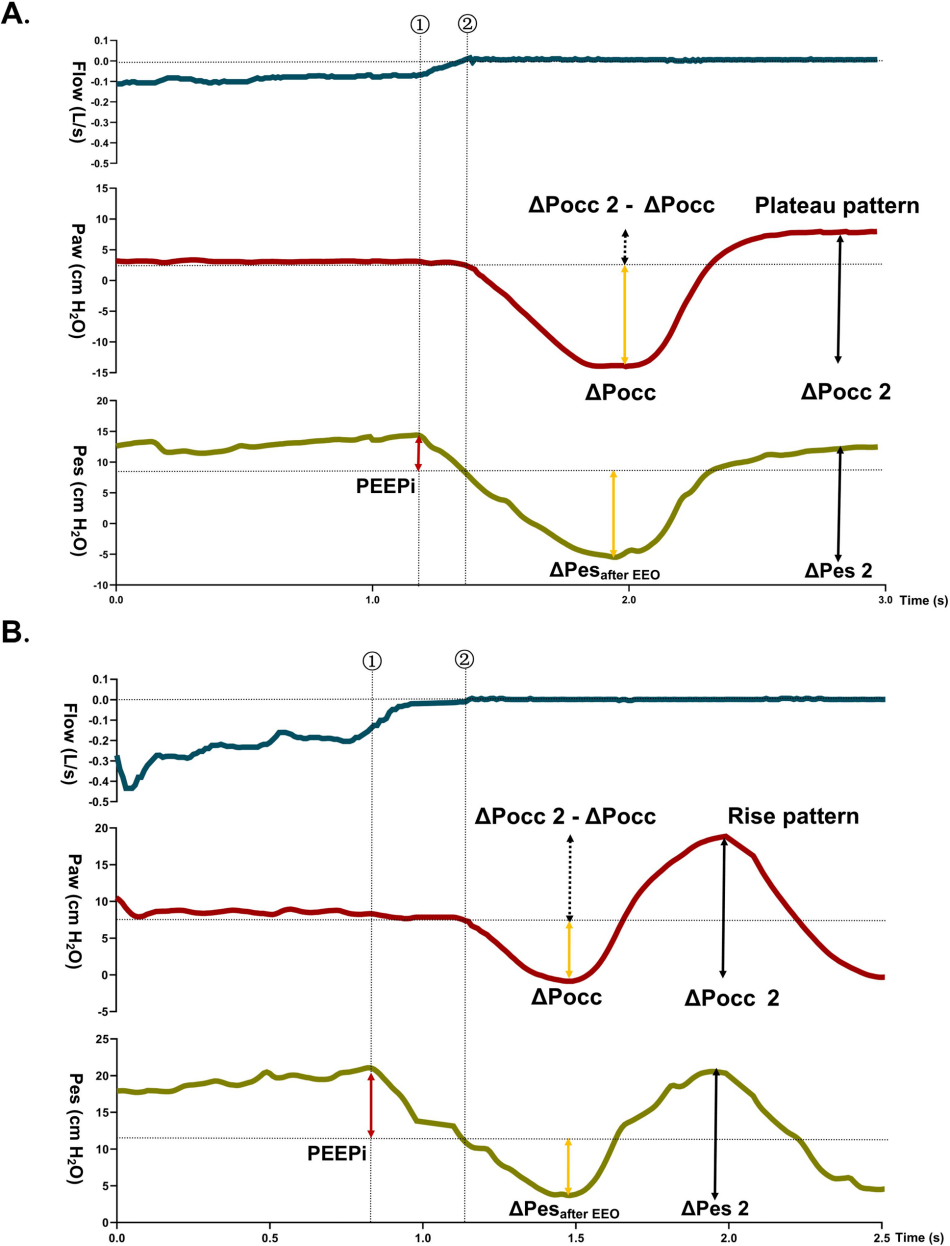
Interpretation of ΔPocc and PEEPi in two patterns. Representative tracing of Flow, airway pressure (Paw), esophageal pressure (Pes) from top to bottom. Dashed lines indicate: ① onset of inspiratory effort, ② end-expiratory occlusion. Baydur’s Ratio is calculated as ΔPocc / ΔPes, whereas the modified Ratio 2 uses ΔPocc / ΔPes_after EEO_ to eliminate PEEPi-related bias, the modified Ratio 3 uses ΔPocc2 / ΔPes2 to incorporate PEEPi-related influences. Panel **A**: The difference between the maximum upward Paw deflection and ΔPocc during EEO (ΔPocc2 - ΔPocc) could be used to estimate PEEPi in **Plateau pattern**. In this case, PEEPi is **5.3** cmH_2_O, (ΔPocc2 - ΔPocc) is **4.8** cmH_2_O; Baydur’s Ratio 1 is **0.8** (14.1 / 17.7 cmH_2_O), Modified Ratio 2 is **1.1** (14.1 /12.4 cmH_2_O), and Modified Ratio3 is **1.1** (18.9 / 17.4 cmH_2_O). Panel **B**: (ΔPocc2 - ΔPocc) cannot accurately estimate PEEPi in **Rise pattern**. In this case, PEEPi measured from Pes is 7.2 cmH_2_O, and PEEPi measured from Pdi is **4.1** cmH_2_O, however, (ΔPocc2 - ΔPocc) is **10.1** cmH_2_O; Baydur’s Ratio 1 is **0.5** (7.7 / 14.2 cmH_2_O), Modified Ratio 2 is **1.1** (7.7 /7.0 cmH_2_O), and Modified Ratio3 is **1.1** (17.8 / 16.9 cmH_2_O)

1) ΔPocc is calculated as the first maximal negative airway pressure (Paw) deflection during an EEO. After reaching this trough, Paw increased toward baseline during relaxation. The maximal upward Paw deflection after the trough was defined as ΔPocc2. The 61 EEOs waveform were classified into two patterns by evaluating changes in the Paw slope following ΔPocc based on predefined criteria: visible plateau or unstable rise (Fig. 2 and S2). Statistical classification required that traces with any uncertainty regarding a stable plateau were conservatively assigned to the “rise pattern”. *Reference* PEEPi was then compared with (ΔPocc2 – ΔPocc) for these two patterns.

2) ΔPocc is used to verify the position of the esophageal balloon during spontaneous breathing by assessing the ratio of ΔPocc to the corresponding Pes swing [8]. It is unclear how is the Ratio measured in case of PEEPi. We calculated the Ratio in three ways: Ratio 1 is ΔPocc to ΔPes swing during EEO (Baydur’s maneuver); Ratio 2 is ΔPocc to the corresponding downward Pes deflection after EEO (ΔPes_after EEO_), and Ratio 3 is the ratio of ΔPocc2 to the corresponding upward Pes deflection (ΔPes2) (Fig. 2).

3)ΔPocc has been proposed for predicting trans pulmonary pressure (*predicted* ΔP_L, dyn_) using predicted ΔPes [4, 13]. The *reference* ΔP_L, dyn_ is the dynamic difference between Paw and Pes [14].

$$Predicted\,\, \Delta P_{L, \rm dyn}\,=\,\Delta \rm Paw\,+\,0.66\,\times\,\Delta Pocc$$

### Statistics

We used linear mixed-effects models to compare the two variables across different PEEP and PEEPi levels, including their interaction as fixed effects and subject as a random intercept. Within- and between-subject components of PEEPi were separated using the Mundlak adjustment. Overall effects were tested using Wald χ² statistics, and post-hoc contrasts were Holm-adjusted for multiple comparisons. Subjective inter-observer agreement was quantified using Fleiss’ kappa coefficient and overall percentage agreement. Bias and agreements were assessed using Bland - Altman analysis. All analyses were performed in Python (version 2025.2.3), with *P* < 0.05 considered statistically significant.

## Results

ΔPocc was missing at two PEEP levels, leaving a total of 61 PEEP-level observations analyzed. Pga was not recorded in three patients, and Pdi was available for 40 PEEP levels.

### PEEPi changes induced by PEEP

**Fig. 3:**
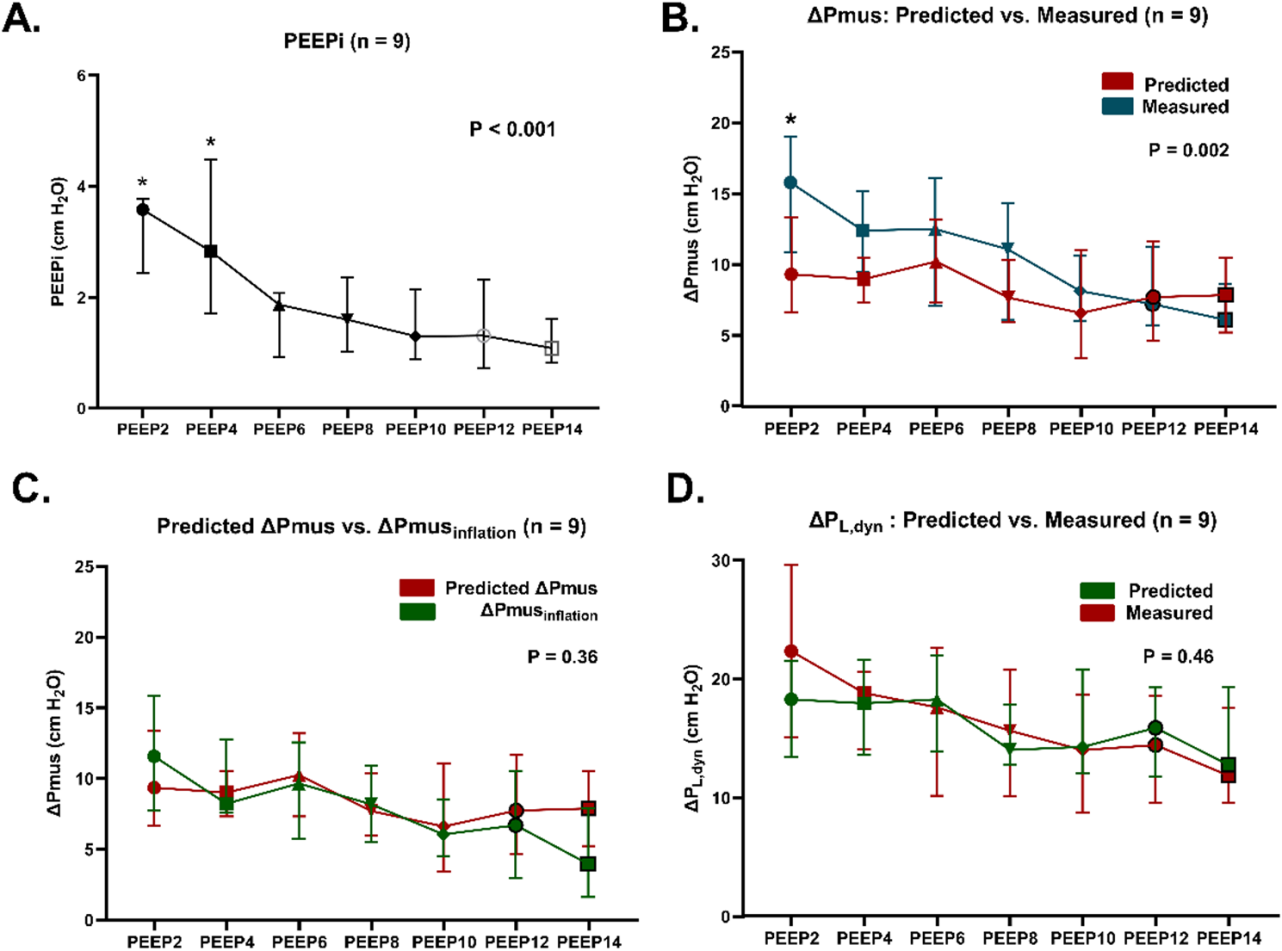
Effects of PEEP on PEEPi, the relationship between *predicted* ΔPmus and *reference* ΔPmus and ΔPmus_inflation_ and the relationship between *predicted* ΔP_L, dyn_ and *reference* ΔP_L, dyn_. Panel **A**: Changes of PEEPi induced by changing PEEP levels, as estimated by Pes changes before inflation. Data are shown as Mean ± SD. Panels **B**: Relationship between *reference* ΔPmus and *predicted* ΔPmus. Across PEEP levels, *predicted* ΔPmus was significantly lower than *reference* ΔPmus (*P* = 0.002). Panel **C**: Relationship between ΔPmus_inflation_ and *predicted* ΔPmus. *Predicted* ΔPmus was similar to ΔPmus_inflation_ at any PEEP level (*P* > 0.05). Panel **D**: Relationship between *predicted* ΔP_L, dyn_ and *reference* ΔP_L, dyn_. *Predicted* ΔP_L, dyn_ was similar to *reference* ΔP_L, dyn_ at any PEEP level (*P* > 0.05). Data are shown as Median and IQR in Panel B, C and D. * represents post-hoc P value is significant

PEEPi values measured by ΔPes_before inflation_ ranged from mean (± SD) of 4.7 (± 5.0)cmH_2_O at PEEP 2 to 1.2 (± 0.7)cmH_2_O at PEEP14 in the 9 patients. At PEEP 8 and PEEP 10, the mean PEEPi was around 2 cmH_2_O (Fig. 3A). The PEEPi measured both by ΔPes_before inflation_ (*n* = 9 patients, Fig. 3A) and by ΔPdi_before inflation_ (*n* = 6 patients, *Figure S1A*) decreased with increasing PEEP (*P* < 0.001 for both, mixed-effects model). From PEEP 6 cmH₂O and above, ΔPes_before inflation_ (and ΔPdi_before inflation_) decreased compared with lower levels (Holm-adjusted *P* < 0.05 for each). ΔPes_before inflation_ was higher than ΔPdi_before inflation_, mostly because of one patient (*P* = 0.002).

### Endpoints

#### Relationship between ΔPocc and ΔPmus

Reference ΔPmus was higher than predicted ΔPmus in the 9 patients with Pes monitoring (median (IQR) of 9.7 (6.4, 13.0) vs. 8.3 (6.3, 11.4), P = 0.002, Figure 3B), with a significant difference observed at PEEP 2 cmH₂O (Holm-adjusted P < 0.05). Similarly, reference ΔPmus was also higher than predicted ΔPmus in the 6 patients with Pga monitoring (median (IQR) of 11.2 (6.9, 15.4) vs. 7.4 (5.3, 11.2) cmH2O, P < 0.001, Figure S1B). When considering only ΔPmus_inflation_ predicted ΔPmus was similar to ΔPmus_inflation_ (8.3 (6.3, 11.4) vs. 8.1 (4.7, 11.1) cmH2O in 9 patients with Pes and 7.4 (5.3, 11.2) vs. 8.5 (6.3, 12.2) cmH2O in 6 patients with Pga respectively, P > 0.05, Figure 3C and S1C). 

#### Using ΔPocc to assess PEEPi

Among the 61 analyzed EEO waveforms, complete agreement (all 4 reviewers) was achieved in 42 cases (68.9%), majority agreement (≥3 reviewers) in 57 cases (93.4%), and 2-2 split in only 4 cases (6.6%). The overall inter-observer agreement was 87.5% with a Fleiss' kappa of 0.72 (good agreement): 52.5% of waveform were classified in Plateau pattern, whereas 47.5% in Rise pattern (Figure S2). Bland–Altman analysis showed that, in the plateau pattern, the bias (95% LOA) between (ΔPocc2 - ΔPocc) and reference PEEPi was 0.78 (-2.31, 3.85) cmH₂O (measured by Pes) and 0.99 (-2.18, 4.16) cmH₂O by Pdi. In the rise pattern, the bias (95% LOA) was 3.76 (10.46, -2.94) cmH₂O by Pes and 7.54 (-8.26, 23.34) cmH₂O by Pdi (Figures S3).

#### Using ΔPocc to calibrate esophageal balloon position

The mixed-effects model showed that Ratio 1 was significantly lower than both Ratio 2 (reference) and Ratio 3 across all PEEP levels (P < 0.001, Figure S4). In contrast, no significant difference was observed between Ratio 2 and Ratio 3 in both plateau pattern and rise pattern (P > 0.05), with a bias (95% LOA) of -0.04 (-0.63 to 0.54). 

#### Using ΔPocc to predict ΔPL,dyn

The mixed-effects model showed that no significant difference was observed between the predicted ΔPL,dyn and the reference ΔPL,dyn across all PEEP levels (Figure 3D) with bias (95% LOA) of 0.5 (-11.4, 12.4) cmH2O.

## Discussion

This study examined whether ΔPocc quantification included the part of the inspiratory effort required to overcome PEEPi during PSV. The main findings are that (1) ΔPocc does not represent the total inspiratory effort when PEEPi is present; (2) Baydur’s maneuver for esophageal balloon calibration might be also biased; (3) when the airway pressure following ΔPocc exhibits a visible stable plateau, PEEPi may be estimated under specific waveform conditions on the ventilator, in selected patients; (4) the predicted estimate of ΔP_L, dyn_ appears acceptable at group level although it can be insufficiently precise individually.

### Pre-inflation component of effort

Previous studies performed in patients with hypoxemic respiratory failure evaluating ΔPocc did not report the presence or magnitude of PEEPi, presuming that intrinsic PEEP was likely minimal in those cohorts [2, 10]. Consistent with recent epidemiological data, high PEEPi values have become less common in invasively ventilated patients [15, 16], although it warrants continued attention. ΔPes_before inflation_ was present in our selected population. When active expiration occurs, Pga drop at end-expiration (i.e., relaxation of the abdominal muscles) may be transmitted to the Pes drop, therefore ΔPes_before inflation_ cannot always be considered synonymous with PEEPi related to hyperinflation. The Pga drop was substantial in only one patient. ΔPdi_before inflation_ provides a more accurate estimate of the load due to hyperinflation because it subtracts the drop in Pga concomitant with the drop in Pes. EEOs start when the expiratory flow has reached zero and ΔPocc is inherently not synchronized with the pre-inflation effort. Therefore, ΔPocc is not directly impacted by any Pes/Pga change happening before occlusion.

### Noninvasive assessment of PEEPi during spontaneous breathing

The feasibility of estimating PEEPi during EEO was further explored. A stable Paw plateau during EEO reflects total PEEP and is used to quantify PEEPi during passive ventilation. In our spontaneously breathing patients, 53% of the Paw pattern following ΔPocc exhibited a visible stable plateau, (ΔPocc2 - ΔPocc) appeared to approximate PEEPi, with light overestimation. In contrast, when Paw following ΔPocc showed an unstable rise, noninvasive estimation of PEEPi in spontaneous breathing was massively influenced by expiratory muscle activity. This waveform categorization was based on a reproducible classification framework with good inter-reviewer agreement according to our analysis. This noninvasive estimation strategy cannot be generalized across all patients receiving PSV.

### Implication for esophageal balloon calibration

Our findings indicate that standard esophageal balloon calibration based on ΔPocc might be biased by PEEPi or active expiration. Baydur’s maneuver assumes that Paw and Pes swings during an occlusion reflect the same inspiratory effort, with a ratio of 0.8–1.2 considered acceptable [8]. We explored two modified ratios to correct this inference: by restricting analysis to the downward Pes deflection occurring after EEO, Ratio 2 isolates the effort component aligned with ΔPocc, alternatively, Ratio 3 incorporates all PEEPi-related influences with easier manipulation. These alternative approaches require prospective validation.

### Estimating dynamic trans-pulmonary pressure

ΔPaw and the maximum ΔPes are often not perfectly synchronous (Pes reaches its minimum before Paw reaches its peak) during inspiration, which leads ΔPaw + ΔPes to overestimate the *reference* ΔP_L, dyn_. ΔPocc underestimates ΔPes in this setting. Finally, the underestimation from 0.66 × ΔPocc seems to offset the overestimation inherent in ΔPaw + ΔPes. ΔPocc may thus provide an acceptable estimate of the *reference* ΔP_L, dyn_ in the presence of PEEPi. Nevertheless, given the small sample size and the wide limits of agreement, prospective validation is warranted. This compensation may diminish when PEEPi becomes substantial, since ΔPocc may underestimate ΔPes substantially.

### Limitations

This study is retrospective and based on a small cohort of patients with PEEPi, and high levels of PEEPi were infrequent even in this population. Because of incomplete Pga/Pdi availability, our findings have limited generalizability.

## Conclusion

This proof-of-concept physiological study shows that ΔPocc can underestimate inspiratory effort in case of PEEPi. When a plateau airway pressure can be observed at the end of ΔPocc, it seems possible to estimate PEEPi noninvasively. The predicted ∆P_L, dyn_ is potentially acceptable but lacks precision in the individual level.

## Supplementary Material

Supplementary Material 1.

## Data Availability

The datasets used and/or analyzed during the current study are available from the corresponding author on reasonable request.

## Abbreviations

PEEPi: Intrinsic positive end-expiratory pressure
PSV: Pressure support ventilation
ΔPocc: End-expiratory whole-breath airway occlusion pressure
Paw: Airway pressure
Pes: Esophageal pressure
Pga: Gastric pressure
Pdi: Transdiaphragmatic pressure
Plung: Transpulmonary pressure
Pmus: Respiratory muscle pressure
P_L,dyn_: Dynamic transpulmonary pressure
EEO: End-expiratory occlusion
