# Supplementary material for: Inspiratory effort estimated by airway occlusion pressure in the presence of intrinsic positive end-expiratory pressure
Source: Crit Care. 2026 Apr 22;30:295. doi: 10.1186/s13054-026-06039-0 (PMC13238042; doi:10.1186/s13054-026-06039-0)
Supplement: Supplementary file 1 — Supplementary Material 1. [file 13054_2026_6039_MOESM1_ESM.docx]

**Supplemental:**

**Inspiratory Effort Estimated by Airway Occlusion Pressure in the presence of Intrinsic Positive End-Expiratory Pressure**

Ran Gao ^1,2,3 #^, Mattia Docci ^1,2,4 #^, Andrea Coppadoro ^5^, Roberto Brito ^1,2,6^, Ewan C. Goligher ^2,7,8,9^, Giacomo Bellani ^10,11^, Laurent Brochard ^1,2,4^*

^1^ Keenan Centre for Biomedical Research, Li Ka Shing Knowledge Institute, Unity Health Toronto, Toronto, Ontario, Canada

^2^ Interdepartmental Division of Critical Care Medicine, University of Toronto, Toronto, ON, Canada

^3^ Surgical Intensive Care Unit, Emergency and Critical Care Medical Center, Clinical and Research Center on Acute Lung Injury, Beijing Shijitan Hospital, Capital Medical University, Beijing, China

^4^ Critical Care Department, St. Michael’s Hospital, Unity Health Toronto, Toronto, Ontario, Canada

^5^ Anesthesia and Critical Care Department, IRCCS San Gerardo, Monza, Italy

^6^ Departamento de Medicina Interna Norte, Facultad de Medicina, Universidad de Chile, Santiago, Chile

^7^ Department of Physiology, University of Toronto,Toronto, Ontario, Canada;

^8^ Division of Respirology, Department of Medicine, University Health Network, Toronto, Ontario,Canada;

^9^ Toronto General Hospital Research Institute, Toronto, Ontario, Canada;

^10^ Centre for Medical Sciences-CISMed, University of Trento, Trento, Italy;

^11^ Department of Anesthesia and Intensive Care, Santa Chiara Hospital, Trento, Italy

^#^ Co-first authorship

^*^ Corresponding author

Laurent Brochard

Keenan Research Centre - St. Michael’s Hospital – Unity Health Toronto

Interdepartmental Division of Critical Care, University of Toronto

Li Ka Shing Knowledge Institute, 4th Floor, Room 411

209 Victoria Street, Toronto, ON M5B 1T8

T: 416 864 5686

F: 416 864 5698

E: [Laurent.Brochard@unityhealth.to](mailto:Laurent.Brochard@unityhealth.to)

Table S1: Clinical data of the population from original EAdi-PEEPi project

| Clinical data | N = 10 |
| --- | --- |
| Age | 74 (75, 78) |
| SAPS II | 47±14 |
| Male (n, %) | 4 (40%) |
| Previous pulmonary conditions (n, %) |  |
| COPD | 5 (50%) |
| Active smoke | 2 (20%) |
| Silicosis | 1 (10%) |
| None | 2 (20%) |
| ICU admission diagnosis (n, %) |  |
| COPD exacerbation | 4 (40%) |
| Sepsis | 3 (30%) |
| Postoperative | 2 (20%) |
| Trauma | 1 (10%) |
| ICU survivors (n, %) | 8 (80%) |
| PaO_2_/FiO_2_ (mmHg) | 222±62 |
| PaCO_2_ (mmHg) | 52.5±14.8 |
| Clinical PEEP (cmH_2_O) | 9±3 |
| Clinical Pressure Support (cmH_2_O) | 4 (6, 12) |
| Days of intubation | 4 (6, 8) |
| Respiratory system compliance (ml/cmH_2_O) | 43±15 |
| Respiratory system resistance (cmH_2_O/L/s) | 17±4 |
| Respiratory system time constant (s) | 0.71±0.30 |

Data are represented as median (interquartile) or mean±SD.

SAPS=simplified acute physiological score; COPD = chronic obsessive pulmonary disease; ICU= intensive care units; PEEP= positive end-expiratory pressure.

Figure S1: Changes of PEEPi and the relationship between *predicted* ΔPmus and *reference* ΔPmus / ΔPmus_inflation_ in six patients with gastric pressure monitoring


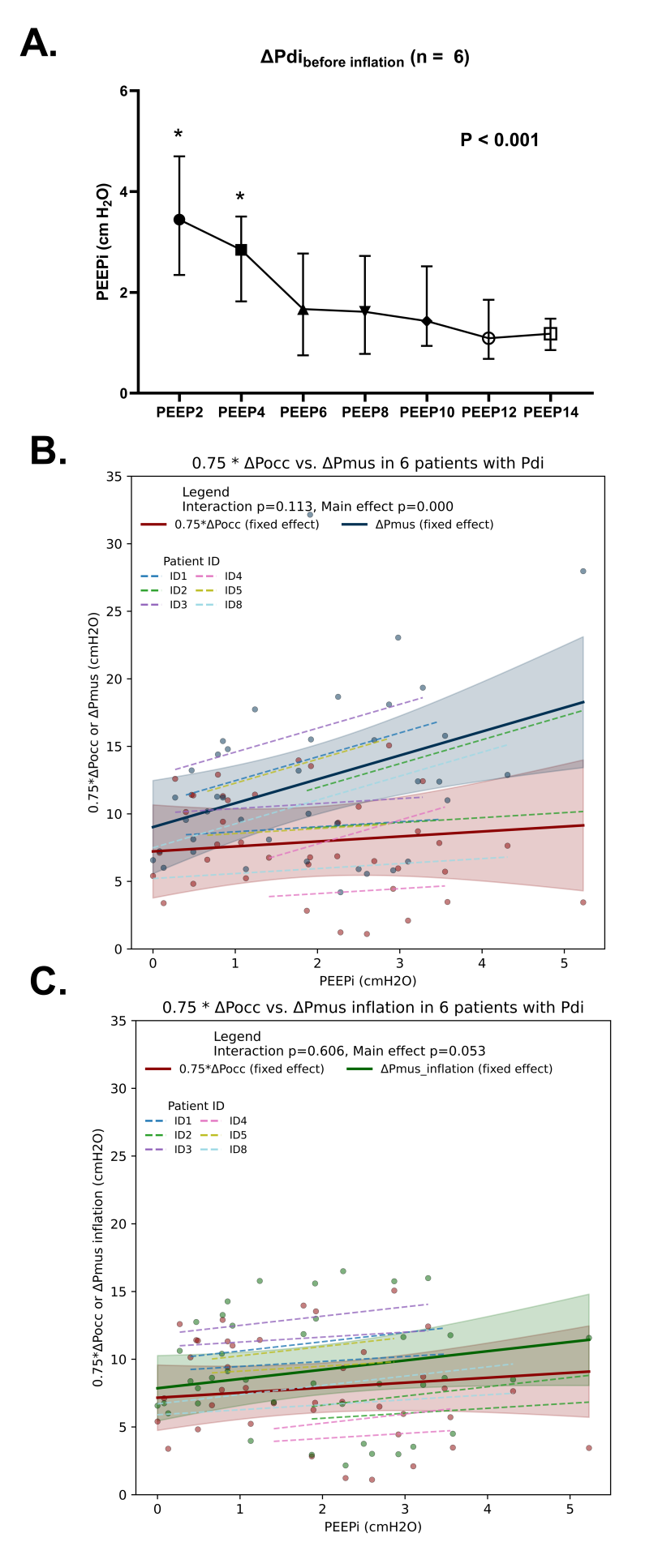


Panel A: Changes of PEEPi induced by changes in different PEEP settings. PEEPi was calculated as ΔPdi_before inflation_, corrected for expiratory muscle relaxation. The mixed-effects model revealed a significant overall effect of PEEP on PEEPi (P < 0.001).

Panels B: Relationship between 0.75×ΔPocc and *reference* ΔPmus across PEEPi levels. ΔPmus remained consistently higher than 0.75 × ΔPocc (P < 0.001).

Panels C: Relationship between 0.75×ΔPocc and ΔPmus_inflation_ across PEEPi levels. In contrast, ΔPmus_inflation_ did not differ significantly from 0.75 × ΔPocc (P > 0.05).

Data are showed by median and IQR.

Figure S2: The assessment of 61 tracings by four independent reviewers


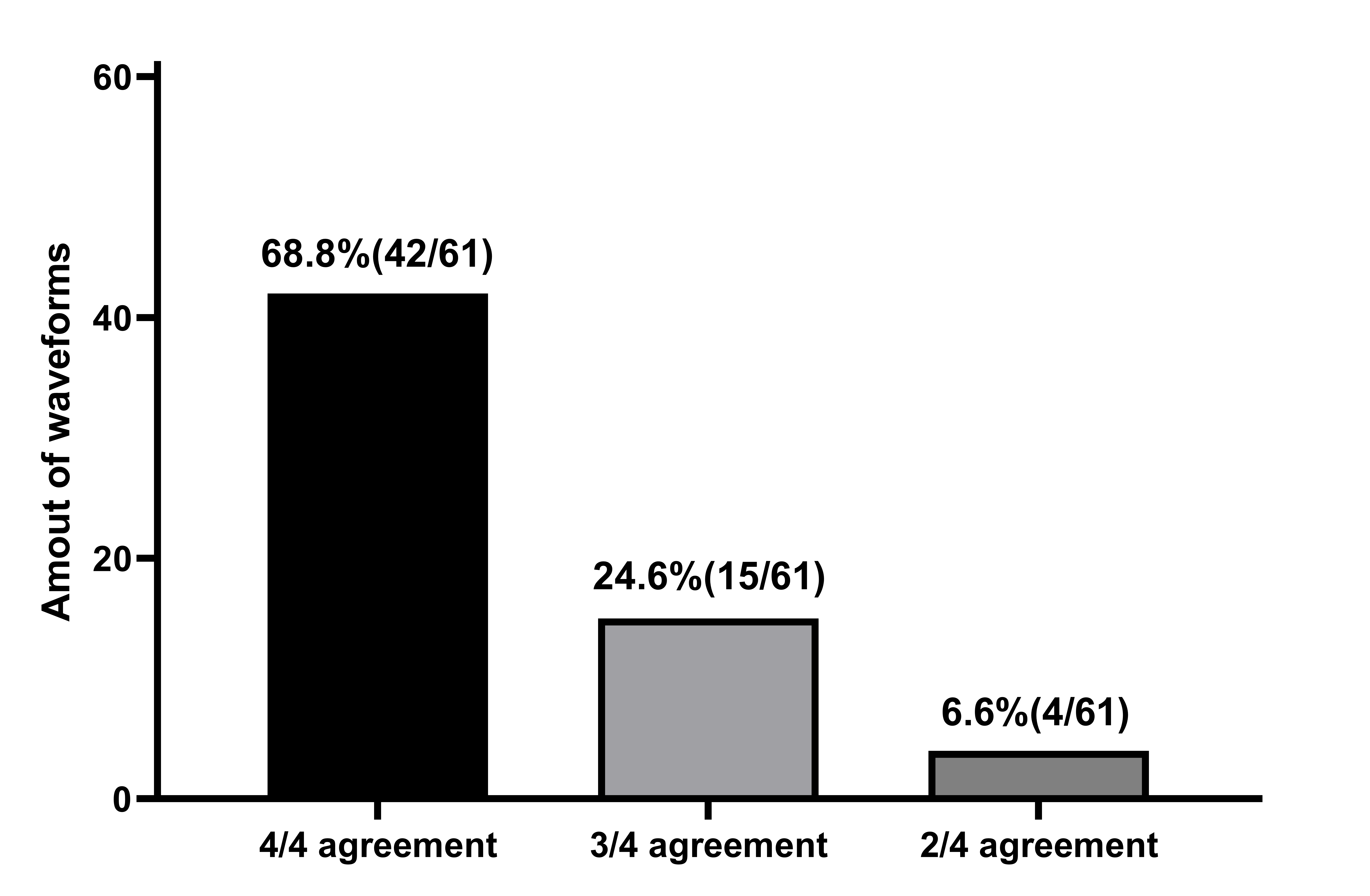


Our approach involved evaluating changes in the Paw slope following ΔPocc. A “plateau pattern” was defined as a clear shift in the Paw slope toward a distinct plateau, indicating respiratory muscle relaxation. All other cases were categorized as a “rise pattern”, including: 1) A continued gentle increase in Paw, suggestive of potential expiratory muscle activity; 2) An initial rise in Paw followed by a sudden drop to a negative slope during the second effort; 3) Other scenarios in which no clear plateau was identified by the reviewer. Four reviewers independently assessed the waveform. Statistical classification required that traces with any uncertainty regarding a stable plateau were conservatively assigned to the “rise pattern”.

After assessment of ΔPocc pattern by four reviewers independently based on the classification criteria, complete agreement (all 4 reviewers) was achieved in 42 cases (68.8%), majority agreement (≥3 reviewers) in 57 cases (93.4%), and 2-2 split in only 4 cases (6.6%), the overall inter-observer agreement was 87.5% with a Fleiss' kappa of 0.72 (good agreement). Considering statistic analysis in a conservative way, 52.5% of waveform were classified into Plateau pattern and 47.5% were classified into Rise pattern in the end to do Bland-Altman agreement with PEEPi reference (Figure S3).

Figure S3: Agreement between PEEPi and (ΔPocc2 - ΔPocc) in two Paw patterns after ΔPocc





Panel A: In 9 patients with Pes, 52.5% (32/61) of breaths shows plateau Paw pattern after ΔPocc. ΔPes_before inflation_ (mean ± SD) in Plateau pattern and Rise pattern were 1.8 ± 1.1 vs. 3.6 ± 4.5 cm H_2_O respectively. Bias (95% LOA) of Plateau pattern vs. Rise pattern is **0.78** (-2.31, 3.85) vs. **3.76** (10.46, -2.94) cmH_2_O.

Panel B: In 6 patients with Pdi, 52% (21/40) of breaths shows plateau Paw pattern after ΔPocc. ΔPdi_before inflation_ (mean ± SD) in Plateau pattern and Rise pattern were 1.57 ± 0.89 vs. 2.56 ± 1.48 cm H_2_O respectively. Bias (95% LOA) of Plateau pattern vs. Rise pattern is **0.99** (-2.18, 4.16) vs. **7.54** (-8.26, 23.34) cmH_2_O.

Figure S4: Comparison between Baydur’s Ratio and Modified Ratio 2 / Ratio 3


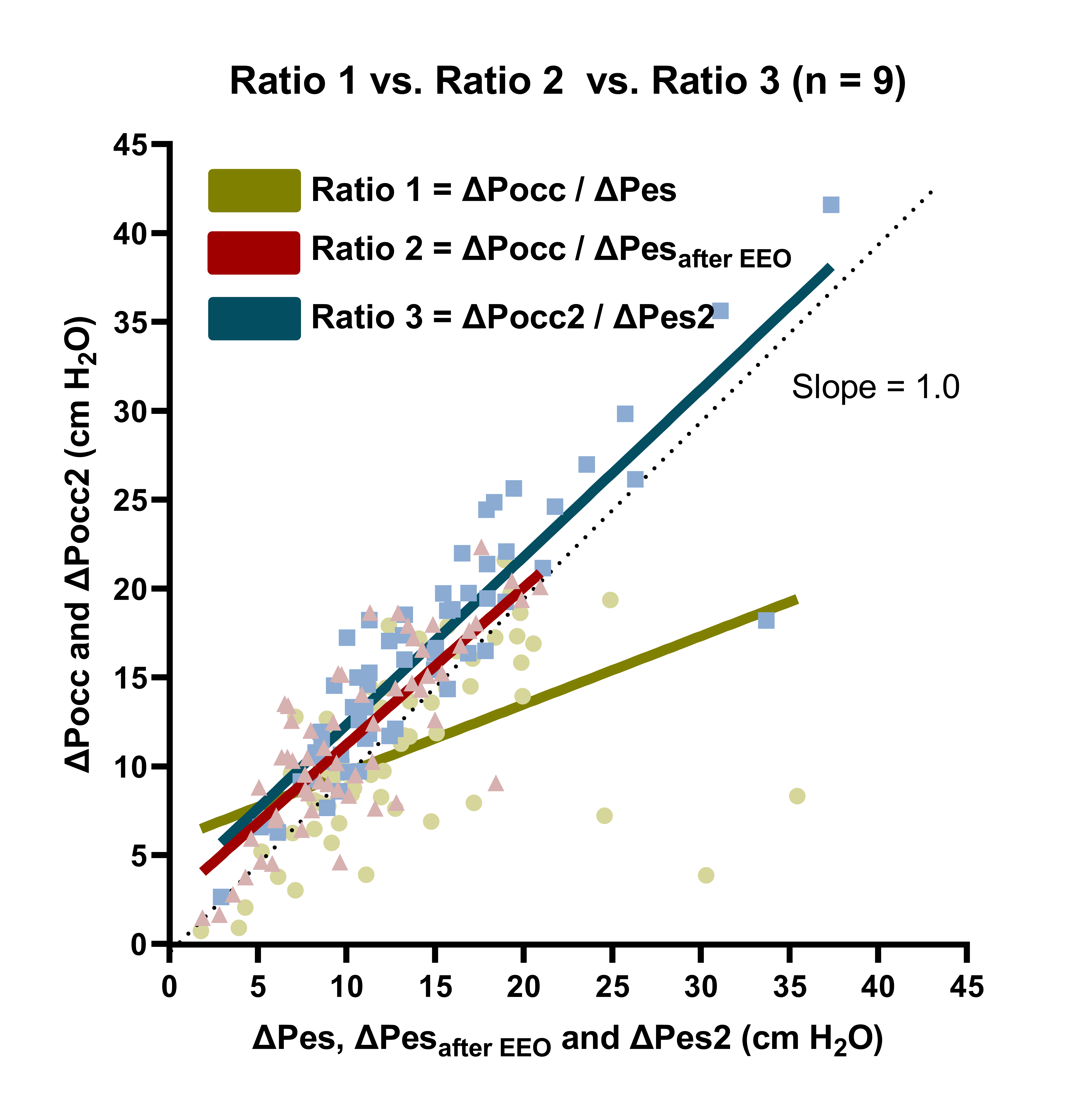


The mixed-effects model showed that Ratio 2 and Ratio 3 were significantly higher than Ratio 1 across all PEEP levels (P < 0.001). No significant difference was observed between Ratio 2 and Ratio 3 (P > 0.05) in both plateau pattern and rise pattern, with a bias (95% LOA) of **-0.04** (-0.63 to 0.54). The slope of each point represents the absolute value of the ratio. It is clear that the slope for Ratio 1 deviates from 1.0, whereas the slopes for Ratio 2 and Ratio 3 are much more similar and closer to 1.0.
